# Prevalence of Clinical and Subclinical *Plasmodium falciparum* and *Plasmodium vivax* Malaria in Two Remote Rural Communities on the Myanmar–China Border

**DOI:** 10.4269/ajtmh.17-0167

**Published:** 2017-09-05

**Authors:** Fang Huang, Shannon Takala-Harrison, Hui Liu, Jian-Wei Xu, Heng-Lin Yang, Matthew Adams, Biraj Shrestha, Gillian Mbambo, Demian Rybock, Shui-Sen Zhou, Zhi-Gui Xia, Xiao-Nong Zhou, Christopher V. Plowe, Myaing M. Nyunt

**Affiliations:** 1 Division of Malaria Research, Institute for Global Health, University of Maryland School of Medicine, Baltimore, Maryland;; 2 National Institute of Parasitic Diseases, Chinese Center for Disease Control and Prevention, Shanghai, China;; 3 World Health Organization Collaborating Centre for Malaria, Schistosomiasis and Filariasis, Shanghai, China;; 4 Laboratory of Parasite and Vector Biology, Ministry of Health, Shanghai, China;; 5 Yunnan Institute of Parasitic Diseases, Pu’er, China;; 6 Department of Geographical Sciences, University of Maryland, College Park, Maryland

## Abstract

Malaria infections may be symptomatic, leading to treatment, or “asymptomatic,” typically detected through active surveillance, and not leading to treatment. Malaria elimination may require purging both types of infection. Using detection methods with different sensitivities, we conducted a cross-sectional study in two rural communities located along the border between China’s Yunnan Province and Myanmar’s Shan and Kachin States, to estimate the prevalence of asymptomatic and symptomatic malaria. In Mong Pawk, all infections detected were asymptomatic, and the prevalence of *Plasmodium falciparum* was 0.3%, 4.3%, 4.0%, and 7.8% by light microscopy, rapid diagnostic test (RDT), conventional polymerase chain reaction (cPCR), and multiplexed real-time PCR (RT-PCR), respectively, and *Plasmodium vivax* prevalence was 0% by all detection methods. In Laiza, of 385 asymptomatic participants, 2.3%, 4.4%, and 12.2% were positive for *P. vivax* by microscopy, cPCR, and RT-PCR, respectively, and 2.3% were *P. falciparum*-positive only by RT-PCR. Of 34 symptomatic participants in Laiza, 32.4% were *P. vivax-*positive by all detection methods. Factors associated with infection included gender (males higher than females, *P* = 0.014), and young age group (5–17 age group compared with others, *P* = 0.0024). Although the sensitivity of microscopy was adequate to detect symptomatic infections, it missed the vast majority (86.5%) of asymptomatic infections. Although molecular detection methods had no advantage over standard microscopy or RDT diagnosis for clinically apparent infections, malaria elimination along the Myanmar–China border will likely require highly sensitive surveillance tools to identify asymptomatic infections and guide targeted screen-and-treat interventions.

## INTRODUCTION

In hopes of preventing the spread of artemisinin-resistant *Plasmodium falciparum* malaria, in 2015 the World Health Organization (WHO) recommended launching a regional campaign to eliminate *P. falciparum* malaria from the Greater Mekong Subregion, which includes Myanmar, Thailand, Cambodia, Vietnam, Laos, and China’s Yunnan Province.^1^ China developed malaria elimination strategies to interrupt local malaria transmission in the entire nation except for the Yunnan–Myanmar border by 2015, and to be completely malaria-free by 2020.^2^ The malaria burden in Yunnan decreased dramatically from 2006 to 2013, but local transmission has persisted, particularly in Menglian and Yingjiang counties that are located adjacent to China’s border with Myanmar’s Shan and Kachin States, respectively. Both *P. falciparum* and *Plasmodium vivax* are present in this region, with *P. vivax* accounting for more than 60% of reported cases in 2014 (National Institute of Parasitic Diseases, Chinese Center for Disease Control Prevention, unpublished data).^3,4^ Transmission is unstable with frequent outbreaks, and up to 50% of malaria cases in Yunnan Province are suspected to be imported from across the long borders with Laos and Myanmar, which have the highest malaria burdens in the region.^5^

Malaria infection can be asymptomatic, or, more accurately, “subclinical,” because subtle symptoms and chronic health effects may occur but not lead to clinical diagnosis and treatment. Malaria elimination may require eradicating both clinically symptomatic as well as these “silent” infections,^6^ which may serve as a reservoir and contribute to ongoing malaria transmission in the face of interventions targeting clinically apparent infections.^7,8^ Although standard diagnostic tests such as light microscopy or rapid diagnostic tests (RDTs) can adequately identify symptomatic infections with high-enough parasite densities, the sensitivity of these tests may be insufficient to detect asymptomatic infections, which can have much lower densities, particularly in low transmission settings such as those found in southeast Asia.^9^ Most studies of asymptomatic malaria infection have used microscopy or RDTs, which have lower limits of detection (LoD) of about 100 parasites/µL (or 10^5^/mL), or more recently, conventional nested polymerase chain reaction (cPCR), with the LoD ranging between 1,000 and 5,000 parasites/mL.^10^ In contrast, a high-volume ultrasensitive PCR assay, using 2 mL of venous blood, has an LoD of 22 parasites/mL^11^ and another finger-prick ultrasensitive PCR assay’s LoD is < 16 parasites/mL,^12^ several 1,000-fold lower than the LoD of standard diagnostic methods. The epidemiology of subclinical malaria has recently been described for sites in Thailand, Cambodia, and Vietnam, where these infections are being targeted by mass drug administration.^9,13^ To expand geographic breadth of our understanding of the epidemiology of subclinical malaria, we conducted a cross-sectional field study to estimate the prevalence of malaria infection in the populations residing at two study sites located on the border between the Yunnan Province of China and northeastern Myanmar’s Shan and Kachin States. We identified malaria infection from a very small volume of blood (40 µL) collected on filter papers using multiplexed real-time PCR (RT-PCR), which has an LoD between those of conventional and ultrasensitive PCR, as well as cPCR, and standard light microscopy and/or RDT.

## METHODS

### Study design and description of study population.

**Figure 1.**
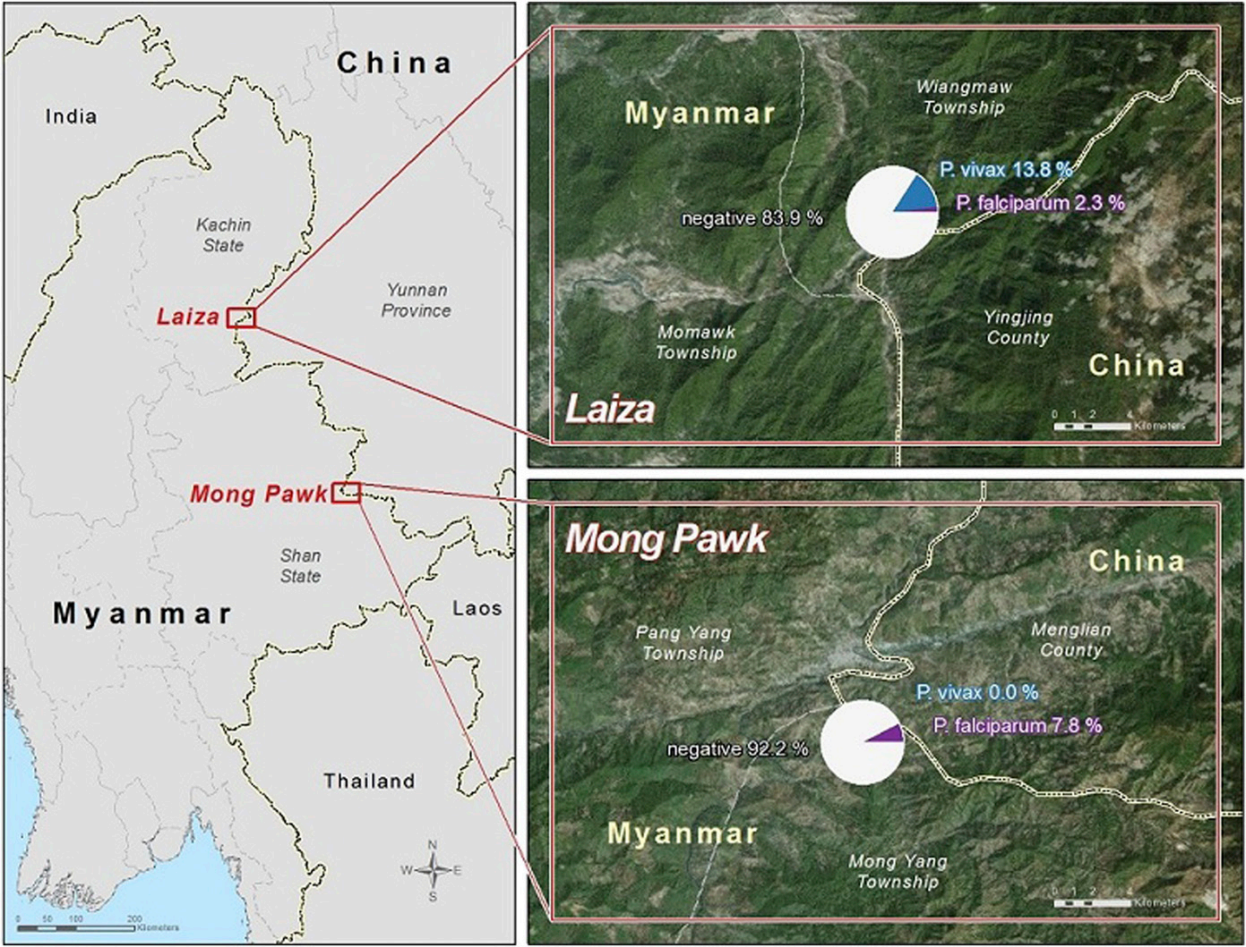
Web-based map of malaria prevalence in study sites. In the inserts showing Mong Pawk and Laiza sites, the white, blue, and purple color in the pie chart represents the proportion of study participants with no, *Plasmodium vivax*, and *Plasmodium falciparum* malaria, respectively. This figure appears in color at www.ajtmh.org.

Cross-sectional community surveys were conducted in June–August 2014 in Mong Pawk and Laiza, two small communities on the Myanmar–China border (Figure 1). Mong Pawk is located in a forested hilly region, about 8 km from the border between Menglian County in China’s Yunnan Province and Shan State of far eastern Myanmar including six communities and the total population is around 12,000. Mong Pawk’s population is composed of highly mobile plantation workers; residents travel frequently (every 3–4 months) through several formal and informal border ports between China and Myanmar. Laiza is situated directly along the border between Yingjiang County of Yunnan Province and Myanmar’s Kachin State. The study population in Laiza was around 10,000 and composed of ethnic refugees who had been living in the Laiza refugee camp for approximately 2–3 years, with limited mobility.

All willing individuals who provided written informed consent were included in the study. Exclusion criteria included age less than 6 months, and history of taking any antimalarial or antimicrobial drugs in the 14 days before enrollment.

### Study procedures.

Participants were recruited by word of mouth (by informing community and religious leaders, and making a verbal announcement about the study at community gatherings and social events). During a specified study period of 3 months, participants were invited and recruited at a small study unit set up at a community center, on a first-come-first-serve basis. Special effort such as recruitment in the evening and on holidays was made to maximize the chance of including participants of all ages, genders, and occupations. Written informed consent was obtained from all adult participants, along with assent and adult guardian consent for children aged < 18 years. Demographic data, including age, gender, and occupation, and medication and travel history were collected. The presence of any symptoms of illness was recorded and axillary temperature taken. Blood was collected from a fingertip or earlobe, for thick and thin smears and RDT (which was available only at the Mong Pawk site) for immediate diagnosis of malaria, and onto filter paper for later PCR analysis. Participants who tested positive for malaria by RDT or microscopy were referred to a malaria clinic affiliated with the study team to receive standard treatment, following the Chinese national malaria treatment guidelines. *Plasmodium vivax* malaria was treated with a total dose of 1,200 mg chloroquine over 3 days, followed by eight daily doses of primaquine 180 mg. *Plasmodium falciparum* malaria was treated with eight tablets of fixed-dose combination of dihydroartemisinin and piperaquine (40 mg/320 mg) for 3 days. Blood collected onto Whatman3MMfilter paper (GE Healthcare Ltd., New Jersey, NJ) were labeled with a unique identification number, air-dried, and individually placed in plastic bags with desiccant, and stored at a room temperature until laboratory analysis.

### Light microscopy and RDT.

Thick and thin blood smears were prepared using Giemsa stain and independently examined under a microscope by two experienced microscopists. One hundred fields of a thick smear were screened before reporting. If the thick smear was positive, 200 fields of a thin smear were screened for species identification. Ten percent of randomly selected negative slides and all positive slides were cross-checked by an independent microscopist as external quality control. All microscopists were certificated as level 1 by WHO monitors. RDTs (SD Malaria Ag *P.f/P.v*; SD Standard Diagnostics, Inc., Suwon, Korea; lot number 145113, expiration date September 9, 2015) were used at the Mong Pawk site, following the manufacturer’s instructions. Targeted antigens in the RDT were *P. falciparum*-specific HRP-2 and *P. vivax*-specific pLDH. The previously reported sensitivities of this RDT in patients with clinical malaria were 99.7% and 95.5% for *P. falciparum* and for *P. vivax*, respectively,^14^ but are unknown in detecting malaria in asymptomatic individuals.

#### Conventional nested PCR.

DNA was extracted from dried blood spots using the QIAamp 96 DNA Blood Kit (Qiagen, Valencia, CA) following the manufacturer’s instructions and the total elution DNA volume for each sample was 100 μL. The cPCR was based on primers targeted to the 18S ribosomal RNA gene described previously.^15^ Briefly, the first amplification reaction used 2 µL of individual DNA in a 20-µL reaction mixture (0.25 mM dNTP, 10 mM Tris-HCL, 30 mM KCl, 1.5 mM MgCl_2_, and 1.0 unit of Taq polymerase containing 0.02 µM primers.) The second amplification was accomplished by using 2 µL of the first PCR product as a template under the same 20-µL reaction mixture conditions. The reaction conditions were as follows: 95°C for 5 minutes, 30 cycles of 95°C for 30 seconds, 55°C for 1 minute, and 72°C for 2 minutes, followed by a single 60°C elongation step for 10 minutes. Amplified products were visualized in 2% agarose gels stained with ethidium bromide. The expected sizes of the *P. falciparum* and *P. vivax* PCR products were 206 and 121 base pair, respectively.

### Multiplexed real-time PCR.

RT-PCR was performed under universal conditions (1 cycle of 50°C for 2 minutes, 1 cycle of 95°C for 10 minutes, and 40 cycles of 95°C for 15 seconds and 60°C for 1 minute) with the ABI 7500 Real Time system. The reaction was performed with a final volume of 25 µL containing 5 µL of DNA, 12.5 µL of TaqMan universal master mix (Applied Biosystems, Torrance, CA), primers, and probes. The primers and probes sequences were published previously,^16^ with fluorophores and concentration modified for this study. The primer and probe with the respective concentrations for each reaction are shown in Table1. Probes were synthesized by Integrated Device Technologies. The *Plasmodium* species of each sample was determined with species-specific forward primers, Plasmo2, and species-specific probes. The reaction was performed in a single tube with distinct fluorophores for each probe. The threshold for *P. falciparum* was 0.1 relative fluorescence units (RFU) at 40 cycles, whereas for *P. vivax* it was set to be 0.04 RFU at 40 cycles. A negative control consisting of the DNA from a negative sample was included in the test panel along with 3D7 *P. falciparum* and *P. vivax* genomic DNA, both from the Malaria Research and Reference Reagent Resource, as positive controls.

**Table 1:** Primers and probes used for screening and identification of *Plasmodium* by RT-PCR

Direction	Name	Sequence
Forward primer*	Fal-F	5′-CCGACTAGGTGTTGGATGAAA GTGTTAA-3′
Forward primer*	Viv-F	5′-CCGACTAGGCTTTGGATGAAAGATTTTA-3′
Reverse primer*	Plasmo 2	5′-AACCCAAAGACTTTGATTTCTCATAA-3′
Taqman probe†	Falprobe	5′-(Cy5)-AGCAATCTAAAAGTCACCTCGAAAGATGACT-BHQ-2-3′
Taqman probe†	Vivprobe	5′-(TAMRA)-AGCAATCTAAGAATAAACTCCGAAGAGAAAATTCT-BHQ-2-3′

RT-PCR = multiplexed real-time polymerase chain reaction.

* Concentration 900 nM.

† Concentration 250 nM.

### Genotyping of Kelch13.

Samples testing *P. falciparum*-positive by RT-PCR were genotyped for the Kelch 13 artemisinin resistance marker. A nested PCR amplification method performed following previously reported protocols^17^ with minor modifications. PCR products were purified using filter plates (Edge Biosystems, Gaithersburg, MD). The product was labeled with Big Dye Terminator v3.1 Reaction Kit (Applied Biosystems) before ethanol precipitation and capillary electrophoresis on an ABI 3730XL automatic sequencer as recommended by the manufacturer’s instructions.

### Statistical analysis.

The data were processed, described, and analyzed using Microsoft Excel 2010 (Reston, VA) and SAS 9.3 (Cary, NC). The prevalence of malaria infection was calculated as the proportion of infection-positive participants divided by the number of successfully tested participants using each study method (RDT, microscopy, cPCR, and RT-PCR). Potential risk factors for malaria infection evaluated included study site, age (categorized into < 5, 5–17, 18–40, 41–60, and > 60 years), gender, and travel to areas known or suspected to be malaria endemic. The prevalence of asymptomatic malaria infection within each of these risk categories was calculated and assessed using a χ^2^ test. The independent effect of the risk factors on malaria infection was determined using multivariate logistic regression analysis.

### Ethical consideration.

The study protocol was independently reviewed and approved by the Institutional Review Boards of the National Institute of Parasitic Diseases, Chinese Center for Disease Control and Prevention, and the University of Maryland, Baltimore, MD.

## RESULTS

### Study population characteristics.

A total of 765 participants (346 from Mong Pawk and 419 from Laiza) were enrolled. Of those, 191 (55.2%) and 224 (53.6%) were female in Mong Pawk and Laiza, respectively. The median (range) age of participants was 23.8 years (0.5–80 years) and 23.5 years (0.8–81 years) in the Mong Pawk and Laiza sites, respectively. The age distribution was similar between the two sites (Table 2). All participants from Mong Pawk were asymptomatic, whereas 34 (8.0%) participants from Laiza presented with signs or symptoms including fever of at least 37.5°C, chills, headache, body ache, nausea, fatigue, and/or loss of appetite. All the participants from Mong Pawk were rubber plantation workers or their families, and although they were not permanent residents of the study area, they reported no travel outside of the plantation in the past month. Residents of the Laiza site were primarily refugees, and others included students, farmers, business people, and teachers. Of 419 participants in the Laiza site, 27 (6.4%) reported frequent travel in the past month to Myitkyina, the malaria-endemic capital city of Myanmar’s Kachin State.

**Table 2:** Age distribution of study population in Mong Pawk and Laiza study sites

Age group (years)	Number (%) tested	
	Mong Pawk	Laiza
< 5	53 (15.3)	104 (24.8)
5–17	101 (29.2)	139 (33.2)
18–40	133 (38.4)	72 (17.2)
41–60	46 (13.3)	55 (13.1)
> 60	13 (3.8)	49 (11.7)

### Prevalence of *Plasmodium* infection.

**Figure 2.**
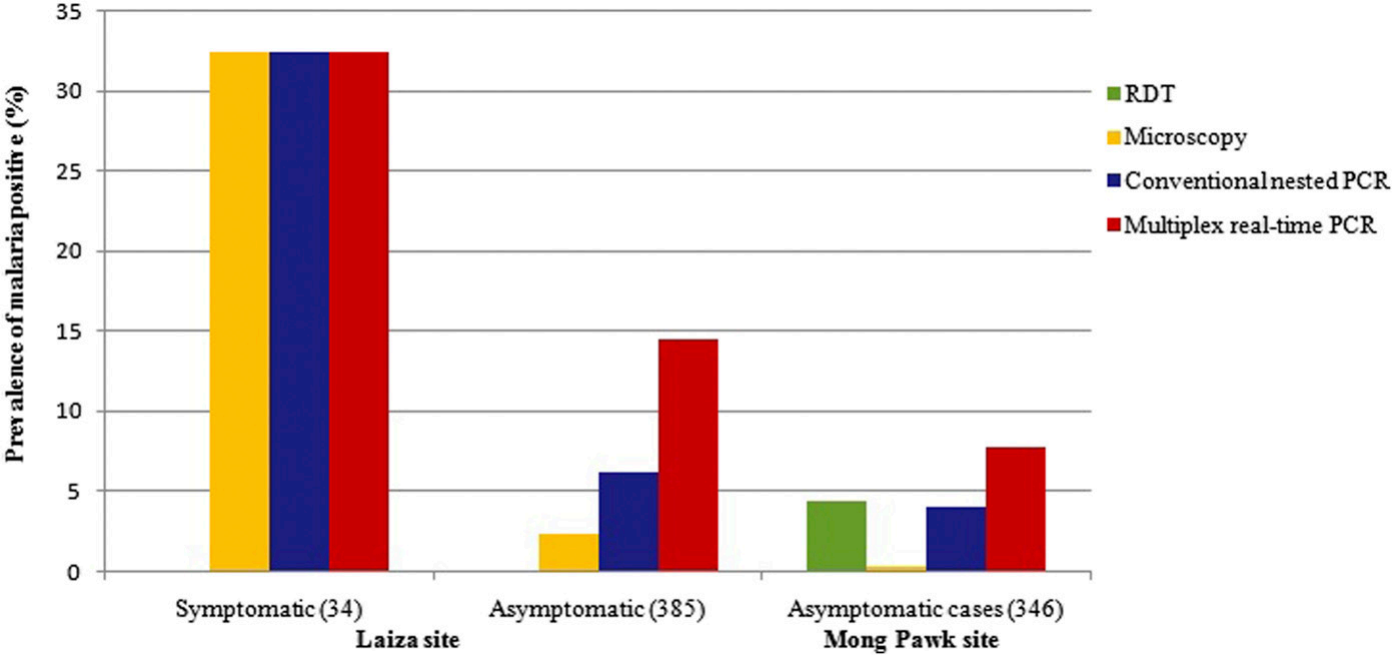
Prevalence of asymptomatic and symptomatic *Plasmodium falciparum* and *Plasmodium vivax* infection detected by microscopy, conventional nested polymerase chain reaction (PCR), and multiplex real-time PCR in study sites. Rapid diagnostic test (RDT) kits were available, and this test was conducted only in Mong Pawk. This figure appears in color at www.ajtmh.org.

The prevalence of *P. falciparum* and *P. vivax* infection, detected by different study methods, is shown in Table 3 and Figure 2. In Mong Pawk, all infections identified were asymptomatic *P. falciparum*—no symptomatic infections and no *P. vivax* infections were detected at this site. Among 346 participants, 1, 15, 14, and 27 infections were identified by microscopy, RDT, cPCR, and RT-PCR, respectively. The prevalence of asymptomatic malaria infection was 7.8% by RT-PCR. Compared with RT-PCR, microscopy missed 96%, RDT missed 44%, and cPCR missed 48% of infections. In Laiza, of 385 asymptomatic participants, 9, 17, and 47 were positive for *P. vivax* by microscopy, cPCR, and RT-PCR, respectively, and 9 were positive for *P. falciparum* infections only by RT-PCR. Among 34 participants who had fever or other symptoms suggestive of acute clinical malaria, none tested positive for *P. falciparum* by any of the study methods, and 11 were positive for *P. vivax* consistently by microscopy, cPCR and real-time PCR. Of the 11 patients (72.7%), eight were of age between 5 and 40 years. RDT testing was not done at this study site. A map of the study site and prevalence of malaria is shown in Figure 1.

**Table 3:** Prevalence of malaria infections detected by microscopy, RDT, cPCR, and RT-PCR

Sites	No. (%) positive							
	*Plasmodium falciparum*				*Plasmodium vivax*			
	Microscopy	RDT	cPCR	RT-PCR	Microscopy	RDT	cPCR	RT-PCR
Mong Pawk (*N* = 346)*	1 (0.3)	15 (4.3)	14 (4.0)	27 (7.8)	0	0	0	0
Laiza (*N* = 419)	0	ND	0	9 (2.3)	20 (4.8)	ND	28 (6.7)	58 (13.8)
Asymptomatic (*N* = 385)	0	ND	0	9 (2.3)	9 (2.3)	ND	17 (4.4)	47 (12.2)
Symptomatic (*N* = 34)	0	ND	0	0	11 (32.4)	ND	11 (32.4)	11 (32.4)

cPCR = conventional nested polymerase chain reaction; ND = not done; RDT = rapid diagnostic test; RT-PCR = multiplexed real-time PCR.

* All participants in Mong Pawk were asymptomatic.

### Concordance between tests.

For symptomatic *P. vivax* infections, the concordance between microscopy, cPCR, and RT-PCR was 100%, with 11 samples being positive for *P. vivax* by all three tests. For asymptomatic *P. vivax*, only nine of 47 RT-PCR-positive infections were microscopy-positive, whereas 8 of these nine (89%) microscopy-positive infections were positive by both cPCR and RT-PCR. All 17 cPCR-positive asymptomatic *P. vivax* infections were also positive by RT-PCR, whereas cPCR missed 30 of 47 (64%) RT-PCR-positive infections.

For *P. falciparum* infections, all of which were asymptomatic, microscopy missed all but one of the 14 cPCR-positive and 27 RT-PCR-positive infections. RDTs missed five of 14 cPCR-positive and 18 of 27 RT-PCR-positive infections, but only nine of the 15 RDT-positive infections were positive by cPCR and RT-PCR. All cPCR-positive *P. falciparum* infections were also positive by RT-PCR, whereas cPCR missed 22 of 36 (61%) RT-PCR-positive *P. falciparum* infections.

### K13 genotyping.

A total of 36 *falciparum*-positive samples detected by RT-PCR were genotyped by capillary sequencing, and 12 of these were successfully sequenced. All of them were found to contain wild-type K13, with no detectable polymorphisms known or suspected to be associated with artemisinin resistance.

### Risk factors for malaria infection.

**Figure 3.**
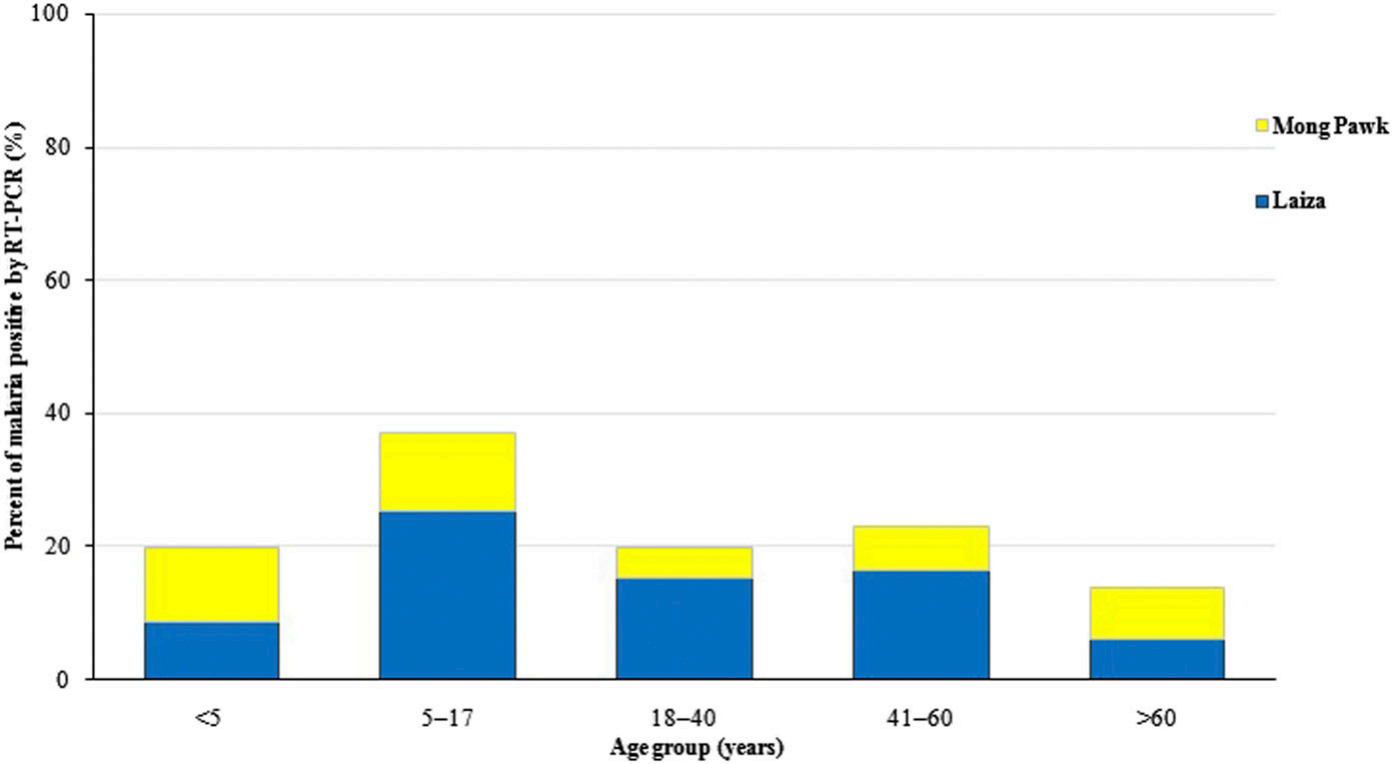
Prevalence of malaria by age group in two study sites. This figure appears in color at www.ajtmh.org.

Study site, gender, age, and travel history were assessed as risk factors for malaria infection determined by RT-PCR (Table 4). In an unadjusted analysis, the proportion of malaria infections was significantly greater at the Laiza than the Mong Pawk site, in males compared with females, in the 5–17 age group than the other age groups (Figure 3), and in those with positive travel history than those without. In an adjusted logistic regression analysis, the log odds of malaria infection was 2.26 times higher in Laiza compared with Mong Pawk (odds ratio [OR] = 2.26, 95% confidence interval [CI] = 1.38–3.69, *P* = 0.0012), 1.76 times higher in males compared with females (OR = 1.76, 95% CI = 1.12–2.75, *P* = 0.014), and 2.71 times higher in the 5–17 age group compared with individuals < 5 years of age (OR = 2.71, 95% CI = 1.42–5.16, *P* = 0.0024). The log odds of malaria infection in the other age groups did not differ significantly from that in individuals aged < 5 years. Travel history was not significantly associated with any malaria infection after adjusting for study site. This is likely because of the fact that all but one of the individuals with a travel history had a symptomatic infection, and all of the symptomatic infections were from the Laiza study site.

**Table 4:** Prevalence of any malaria infection determined by RT-PCR in different risk group

Risk factors	Mong Pawk		Laiza	
	No. tested	No. (%) positive	No. tested	No. (%) positive
Total	346	27 (7.8)	419	67 (16.0)
Gender				
Female	191	10 (5.2)	224	29 (12.9)
Male	155	17 (11.0)	195	38 (19.5)
Age group				
< 5	53	5 (9.4)	104	9 (8.7)
5–17	101	10 (9.9)	139	37 (26.6)
18–40	133	8 (6.0)	72	9 (12.5)
41–60	46	2 (4.3)	55	9 (16.4)
> 60	13	2 (15.4)	49	3 (6.1)
Travel				
Yes	0	0 (0.0)	27	7 (25.9)
No	346	27 (7.8)	392	60 (15.3)

RT-PCR = multiplexed real-time polymerase chain reaction.

## DISCUSSION

This cross-sectional survey revealed the presence of reservoirs of asymptomatic malaria in otherwise generally healthy plantation workers and refugees living in remote rural areas along the border between China’s Yunnan Province and Myanmar’s Shan and Kachin States. The size of this “silent” reservoir was substantially underestimated by standard RDT or microscopy in this study, which detected malaria infection using cPCR and RT-PCR, in order of increasing sensitivity.

In participants with acute clinical malaria at the Laiza site, malaria was detected uniformly by microscopy, cPCR, and RT-PCR (RDT was not available in this study site), suggesting that the sensitivity of standard diagnostic tests is adequate when parasite density is high enough to cause clinically recognizable malaria illness in this setting. The higher sensitivity and lower LoDs of molecular testing thus offered no advantage for diagnosing clinical malaria at this site. However, in participants with subclinical malaria, the majority of the infections detected by RT-PCR were missed by light microscopy, RDT, or even cPCR, likely because of low parasite densities that fell below the LoDs of the less sensitive methods. Unexpectedly, in Mong Pawk, more than one-third of RDT-positive asymptomatic *P. falciparum* infections were negative by both cPCR and RT-PCR. Possible explanations for this discordance include false-positive RDTs or unreported recently treated infections with residual antigenemia after parasite nucleic acids had disappeared.

Others have reported that the prevalence of malaria infections was significantly higher when a highly sensitive real-time PCR method was used.^9,18,19^ Our findings suggested that even conventional or standard PCR, which is commonly used for both research and surveillance, may underestimate the true prevalence of subclinical malaria. We used a low volume of dried blood spot (∼14 μL), which may have further limited the sensitivity of our molecular detection methods. The examination of a high volume (∼2 mL) of blood has been used to measure the prevalence of subclinical malaria along the Thailand–Myanmar border.^11^ We have recently developed an ultrasensitive multiplexed reverse transcriptase PCR method that achieves similarly ultralow LoDs using finger-stick samples and requires no processing or cold chain.^12^ We are adapting these methods for use on dried blood spots, aiming to achieve LoDs lower than those of the RT-PCR we used here, ideally as low as obtained with venous and capillary samples.

Although the point-of-care testing ability offered by microscopy or RDTs is essential for malaria case management, the inability of these tests detect the majority of subclinical infections poses a serious challenge to the success of malaria elimination planned in Greater Mekong Subregion, where malaria transmission is low and where the majority of infections are likely to be present in this silent form.^11,13,19,20^ The contribution of low-density infections that can be detected only by sensitive molecular methods to the risk of transmission is unknown, but presumably the parasites have evolved to persist in this form for their survival benefit. Even if the risk of transmission from low-density infections is low at one time point, it is plausible that these infections fluctuate in their density and ability to generate infectious gametocytes. Limited data from Cambodia suggest that persistent asymptomatic infection is more frequent in vivax (35%) than falciparum (13%) malaria.^21^ Further research is needed to assess the extent to which these very low density *P. falciparum* and *P. vivax* infections contribute to the risk of clinical malaria and/or of transmission to inform elimination strategies in the Greater Mekong Subregion. Understanding these risks is essential for weighing the risks and benefits of interventions needed for malaria elimination in populations with infections that are undetectable by RDT and seen only with ultrasensitive tests.

Malaria epidemiology was different at the two sites. Among rubber plantation workers in Mong Pawk, located in Myanmar’s eastern Shan State, only *P. falciparum* infections—all asymptomatic—were found. Standard RDT surveillance would have completely failed to detect a significant malaria burden at this site. In contrast, refugees in Laiza, in northeastern Kachin State, had predominantly *P. vivax* infections, and a significant minority were experiencing clinical symptoms of malaria. The border of Yunnan Province with Shan and Kachin States is one of the last remaining frontiers of malaria endemicity in China, which is striving for malaria elimination by 2020. The presence of a border-spanning asymptomatic reservoir, acting as the primary driving force for continued transmission, complicates the task of elimination, and the predominance of *P. vivax* in some areas poses the additional challenge or treating relapsing malaria.

Interestingly, our study found a significantly higher malaria prevalence among children. This was surprising in light of the fact that clinical malaria in this region is generally recognized to be more common in young adults with occupational exposure to forest-biting mosquitoes.^9,22^ This unexpected finding suggests that either children are going to where the vectors are (forests, workplaces), or vectors are present where children spend their time (in or near the home or school). At the Mong Pawk plantation site, there was no difference in malaria prevalence between young children and adolescents (data not shown), who spend most of their time at home or school. But the risk was higher in young children compared with adolescents in the Laiza refugee camp. We speculate that daytime malaria transmission may be occurring near the primary school attended by the younger children. Entomological investigation is needed to confirm this speculation.

Malaria remains an important infectious disease in the Greater Mekong Subregion,^23^ where the risk of resurgence is elevated by the presence of multidrug resistance. Myanmar accounts for approximately one-fifth of the region’s population, more than half of the malaria cases and approximately three-quarters of malaria deaths in this region.^24^ Cross-border migration between the Yunnan, China, and Myanmar is thought to be the major source of introduction of *P. falciparum* malaria into southern China.^4^ Malaria outbreaks and re-introduction of malaria in areas previously known to be malaria-free have been frequent in these border areas.^25^ The majority of malaria infections in the Myanmar–China border areas have been determined to be imported from Myanmar, based on travel history. Malaria importation occurs as a result of human activities involving border trade, businesses, border development, and mass population movement due to political instability.^26,27^

As we approach the goal of elimination, malaria parasites can be expected to go to ground, residing at low densities in people and at low prevalence in remote rural locations, especially along border areas that are often beyond the reach of routine health care and surveillance. Molecular epidemiological or seroepidemiological tools that go beyond simply measuring prevalence to estimate parasite migration patterns and identify transmission sources and sinks could further improve our understanding of asymptomatic reservoirs and transmission dynamics in this region, and enhance prospects for elimination.
